# Diffusion tractography reveals pervasive asymmetry of cerebral white matter tracts in the bottlenose dolphin (*Tursiops truncatus*)

**DOI:** 10.1007/s00429-017-1525-9

**Published:** 2017-11-30

**Authors:** Alexandra K. Wright, Rebecca J. Theilmann, Sam H. Ridgway, Miriam Scadeng

**Affiliations:** 1 Center for Marine Biotechnology and Biomedicine, Scripps Institution of Oceanography, University of California-San Diego, La Jolla, CA 92093 USA; 2 Department of Radiology, University of California-San Diego, La Jolla, CA 92093 USA; 3 National Marine Mammal Foundation, San Diego, CA 92106 USA; 4 Center for Functional MRI, Department of Radiology, University of California-San Diego, La Jolla, CA 92093 USA

**Keywords:** Arcuate fasciculus, Asymmetry, Bottlenose dolphin (*Tursiops truncatus*), Diffusion tensor imaging (DTI), Tractography, White matter

## Abstract

Brain enlargement is associated with concomitant growth of interneuronal distance, increased conduction time, and reduced neuronal interconnectivity. Recognition of these functional constraints led to the hypothesis that large-brained mammals should exhibit greater structural and functional brain lateralization. As a taxon with the largest brains in the animal kingdom, Cetacea provides a unique opportunity to examine asymmetries of brain structure and function. In the present study, diffusion tensor imaging and tractography were used to investigate cerebral white matter asymmetry in the bottlenose dolphin (*Tursiops truncatus*). Widespread white matter asymmetries were observed with the preponderance of tracts exhibiting leftward structural asymmetries. Leftward lateralization may reflect differential processing and execution of behaviorally variant sensory and motor functions by the cerebral hemispheres. The arcuate fasciculus, an association tract linked to human language evolution, was isolated and exhibited rightward asymmetry suggesting a right hemisphere bias for conspecific communication unlike that of most mammals. This study represents the first examination of cetacean white matter asymmetry and constitutes an important step toward understanding potential drivers of structural asymmetry and its role in underpinning functional and behavioral lateralization in cetaceans.

**Electronic supplementary material:**

The online version of this article (doi:10.1007/s00429-017-1525-9) contains supplementary material, which is available to authorized users.

## Introduction

Asymmetries of brain structure and function are found throughout the vertebrates (Rogers and Andrew 2002), varying in type and magnitude. An asymmetric or lateralized brain is characterized by anatomical or functional differences between its bilateral components, such as the cerebral hemispheres, cortical areas, or cerebral white matter tracts. It has been hypothesized that the extent of brain lateralization increases with increasing brain size (Ringo 1991; Ringo et al. 1994). This relationship is thought to arise through mechanisms to (1) avoid extreme and untenable brain enlargement consequent to the maintenance of complete neuronal interconnectivity (i.e., the number of neurons to which an individual neuron is directly connected) and (2) mitigate increased interhemispheric conduction delay in large brains resultant from longer transmission distances. The Ringo hypothesis contends that constraints on interconnectivity and conduction time inherent to the evolution of large brains may impose strict limits on global processing and favor local processing of related functions leading to the development of brain lateralization. Studies of cortical arealization (Northcutt and Kaas 1995; Kaas 2013) and hemispheric interconnectivity (Rilling and Insel 1999; Olivares et al. 2000, 2001) provide evidence for enhanced local processing in large-brained mammals and suggest that greater structural and functional lateralization may arise from increased intrahemispheric connections and hemispheric isolation via reduced commissural linkage.

Cetaceans (whales, dolphins, and porpoises) have the largest brains in the animal kingdom (Pilleri and Gihr 1970; Ridgway and Brownson 1984; Ridgway and Tarpley 1996; Ridgway and Hanson 2014). In accordance with the Ringo hypothesis (Ringo 1991; Ringo et al. 1994), a high degree of lateralization would be expected for large cetacean brains. Moreover, deviation from an otherwise evolutionarily conserved cerebral scaling law (Hofman 1989; Wright et al. 2017) in addition to selective pressures of the aquatic environment favoring continuous vigilance (Ridgway et al. 2006a, b, 2009; Branstetter et al. 2012) would be predictive of increased hemispheric lateralization and functional independence of the cerebral hemispheres. Indeed, structural, functional, and behavioral lateralization has been observed throughout the Cetacea, including both the Odontoceti (echolocating toothed whales, dolphins, and porpoises) and Mysticeti (non-echolocating baleen whales). Asymmetry of cortical surface area (Ridgway and Brownson 1984) and subcortical and midbrain structure volumes (Montie et al. 2008; Wright et al. 2017) have been observed in a number of species of the cetacean family Delphinidae. Morphological asymmetry has also been reported for certain midbrain nuclei of balaenopterid mysticetes (Pilleri and Gihr 1970). Behavioral asymmetries indirectly linked to functional lateralization have been widely documented in odontocetes and mysticetes spanning various sensory, motor, cognitive, and social functions (MacNeilage 2013). Arguably, the most striking form of functional lateralization observed in odontocetes is that of unihemispheric slow wave sleep, a state of hemispheric incoherence [i.e., one cerebral hemisphere produces sleeping electroencephalograms (EEGs) while the contralateral hemisphere produces waking EEGs] thought to be important for the maintenance of locomotion, surface respiration, or vigilance toward conspecifics, predators, or prey by one cerebral hemisphere, while simultaneously permitting sleep in the contralateral hemisphere (Supin et al. 1978; Goley 1999; Rattenborg et al. 2000; Ridgway 2002; Lyamin et al. 2008).

Though observations of anatomical, functional, and behavioral asymmetry have been reported in Cetacea, no previous studies have investigated white matter asymmetry in large cetacean brains and its potential functional implications. Therefore, the present study examined the extent of cerebral white matter asymmetry in *Tursiops truncatus*, a delphinid with an average absolute brain size larger than that of *Homo sapiens* and a relative brain size exceeding that of nonhuman anthropoid primates (Ridgway and Brownson 1984; Marino 1998). Diffusion tensor imaging (DTI) and tractography were used for the identification, measurement, and three-dimensional (3D) reconstruction of *T. truncatus* white matter tracts of the association, projection, and commissural fiber systems. The bilateral cerebral white matter tracts of this large *T. truncatus* brain exhibited pronounced lateralization potentially associated with brain enlargement, unique cerebral scaling, or environmental selection pressures. The observation of pervasive asymmetry of the cerebral white matter architecture of *T. truncatus* is proposed to reflect differential perception, processing, and production of social and nonsocial sensory signals and motor actions.

## Materials and methods

### Specimen

The specimen examined was the formalin-fixed brain of a 27-year-old male *T. truncatus* (NAY, body length: 302 cm, body weight: 284 kg). The fresh mass of the specimen was 2093 g. Within 3 h of death, the specimen was extracted, fixed whole in 10% phosphate-buffered formalin, and placed on a shaker to facilitate thorough penetration of the fixative. The specimen was kept for approximately 6 years in regularly changed buffered formalin. The cause of death was phytobezoar asphyxiation and non-neurological in nature.

### Image acquisition and processing

Imaging was conducted at the University of California-San Diego Center for Functional Magnetic Resonance Imaging using a General Electric 3.0 T Signa 750 magnetic resonance imaging (MRI) system with an eight-channel head coil. T2-weighted (fast spin echo, repetition time [TR] = 5500 ms, echo time [TE] = 80 ms, matrix = 384 × 256 [re-gridded onto a 512 × 512 matrix], voxel size = 0.39 × 0.39 × 3 mm, field of view [FOV] = 200 mm, two-dimensional [2D] acquisition with 4 averages, 12 min collection) and T1-weighted (gradient echo, TR = 7.5 ms, TE = 3.2 ms, inversion time [TI] = 400 ms, matrix = 256 × 256, flip angle = 11°, voxel size = 0.78 × 0.78 × 1.2 mm, FOV = 200 mm, 3D acquisition, 15 min collection) high-resolution anatomical images were acquired in the axial plane.

Diffusion tensor images were acquired in the axial plane using a single-shot echo planar imaging (EPI) sequence with diffusion-encoding along 60 directions, *b* value = 3000 s/mm^2^, six non-diffusion-weighted images (*b*_0_), slice thickness = 3 mm, TR = 8 s, TE = 82 ms, 4 averages, matrix = 128 × 128 (automatically re-gridded onto a 256 × 256 matrix), FOV = 200 mm, 56 axial slices, and voxel size 0.78 × 0.78 × 3 mm. The DTI acquisition was repeated three times for a total scan time of 105 min.

DTI data were prepared using FMRIB Software Library (FSL), version 5.0.2.2 (http://www.fmrib.ox.ac.uk/fsl). Images from each DTI acquisition were concatenated (3 total) and corrected for eddy currents using the “eddy” tool provided by FSL. Eddy-corrected DTI acquisitions were then fit to a diffusion model for each voxel using the FMRIB Diffusion Toolbox (FDT; Behrens et al. 2003). The diffusion tensor model was diagonalized to yield the three eigenvalues of the tensor in order to calculate fractional anisotropy (FA), mean diffusivity (*M*_D_), axial diffusivity (*A*_D_), and radial diffusivity (*R*_D_) maps. *R*_D_ maps were calculated as the average of the second and third eigenvalue. The FA and main eigenvector maps were converted and imported into DtiStudio for fiber tracking analysis.

### Tractography and 3D reconstruction

Fiber tracking (i.e., streamline tracking) was performed in DtiStudio (Jiang et al. 2006) using the fiber assignment by continuous tracking (FACT) method (Mori et al. 1999). Tracking was terminated when the local FA fell below the FA threshold of 0.1, or when the tract-turning angle exceeded the angular threshold of 55°. The selected FA threshold exceeded the cerebral gray matter FA of 0.04 ± 0.01 (mean ± standard deviation). The reduced diffusivity and anisotropy of this formalin-fixed specimen (Miller et al. 2011) necessitated a lower FA threshold compared to the default FA threshold (i.e., FA = 0.20) often used for in vivo *H. sapiens* studies.

A multiple region of interest (ROI) approach was used to reconstruct cerebral white matter tracts (Jiang et al. 2006; Wakana et al. 2007). ROIs were identified and manually delineated using FA maps, directionally encoded color maps (red: left–right, green: dorsal–ventral, blue: anterior–posterior), or HSV color images, where appropriate. ROI placement was performed by one author (AKW) and replicated for each tract three times during different sessions on separate days. The ROI protocols implemented for each white matter tract are summarized in the Appendix. 3D volume rendering of the cerebrum and white matter tracts was performed using AMIRA software (FEI Visualization Sciences Group, Burlington, MA, USA).

Eight cerebral white matter tracts of the association, projection, and commissural fiber systems were reconstructed. The association tracts identified included the arcuate fasciculus, cingulum, external capsule, and superior longitudinal fasciculus system. Moreover, the subcomponents of the superior longitudinal fasciculus system (SLF I, SLF II, and SLF III) were identified and isolated. The anterior thalamic radiation, corticocaudate tract (i.e., the white matter of the caudate tail), and fornix comprised the projection tracts isolated. The forceps minor of the corpus callosum was the only commissural tract that could be reliably reconstructed.

### Quantitative analysis

Measurements of the volume (number of voxels containing at least one fiber [i.e., streamline, or reconstructed trajectory] × voxel size; Hagmann et al. 2006), fiber number (number of reconstructed streamlines penetrating the ROI[s]; Jiang et al. 2006), mean fiber length (mean length of reconstructed streamlines; Jiang et al. 2006), FA (degree of anisotropic diffusion; Beaulieu 2014), *M*_D_ (magnitude of diffusivity; Beaulieu 2014), *A*_D_ (parallel diffusivity; Beaulieu 2014), and *R*_D_ (perpendicular diffusivity; Beaulieu 2014) were acquired for each tract. Asymmetries of each tract-specific measurement were assessed by calculating the lateralization index (LI; Vernooij et al. 2007) according to the following equation:

$$ {\text{LI }}\left( X \right) = \left( {X_{\text{Left}} {-}X_{\text{Right}} } \right)/\left( {X_{\text{Left}} + X_{\text{Right}} } \right), $$

where *X* is the tract measurement (e.g., volume or FA). Lateralization index values ranged between −1 and 1. Positive values indicate that tract measurement *X*_Left_ is greater than tract measurement *X*_Right_, whereas negative values indicate that tract measurement *X*_Right_ is greater than tract measurement *X*_Left_. Index values approaching 0 (−0.1 ≤ LI [*X*] ≤ 0.1; Vernooij et al. 2007; Seghier 2008) indicate a comparable tract measurement *X* between the right and left cerebral hemispheres and thus, the absence of asymmetry. Calculations of the relative volume and relative fiber number were performed for each tract to determine the percentage of the total volume or total fiber number occupied by the left and right tracts. Asymmetry of fiber number arises from differences in the number of reconstructed streamlines between hemispheres, whereas tract volume asymmetry is related to differences in the number of voxels containing at least one streamline.

Assessments of asymmetry were not performed for the forceps minor of the corpus callosum or fornix.

## Results

**Fig. 1:**
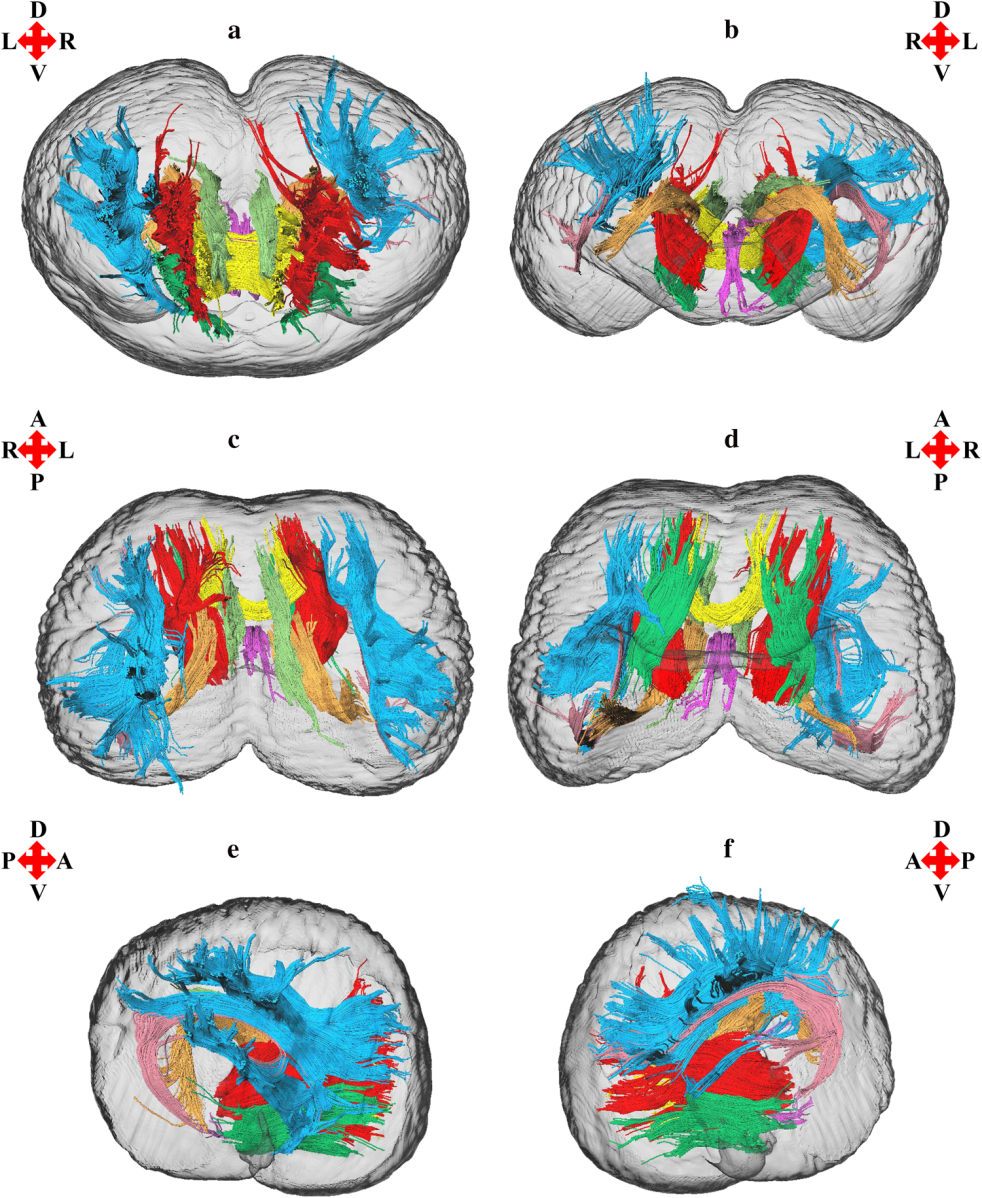
**a** Anterior, **b** posterior, **c** dorsal, **d** ventral, **e** left parasagittal, and **f** right parasagittal views of the *T. truncatus* cerebral surface (translucent dark gray) and underlying white matter tracts of the anterior thalamic radiation (red), arcuate fasciculus (rose), cingulum (light green), corticocaudate tract (orange), external capsule (dark green), forceps minor of the corpus callosum (yellow), fornix (fuchsia), and superior longitudinal fasciculus system (light blue). Color designations are consistent across figures; however, the superior longitudinal fasciculus system in Fig. 3 reflects parcellation of the sub-tracts, SLF I, SLF II, and SLF III

**Fig. 2:**
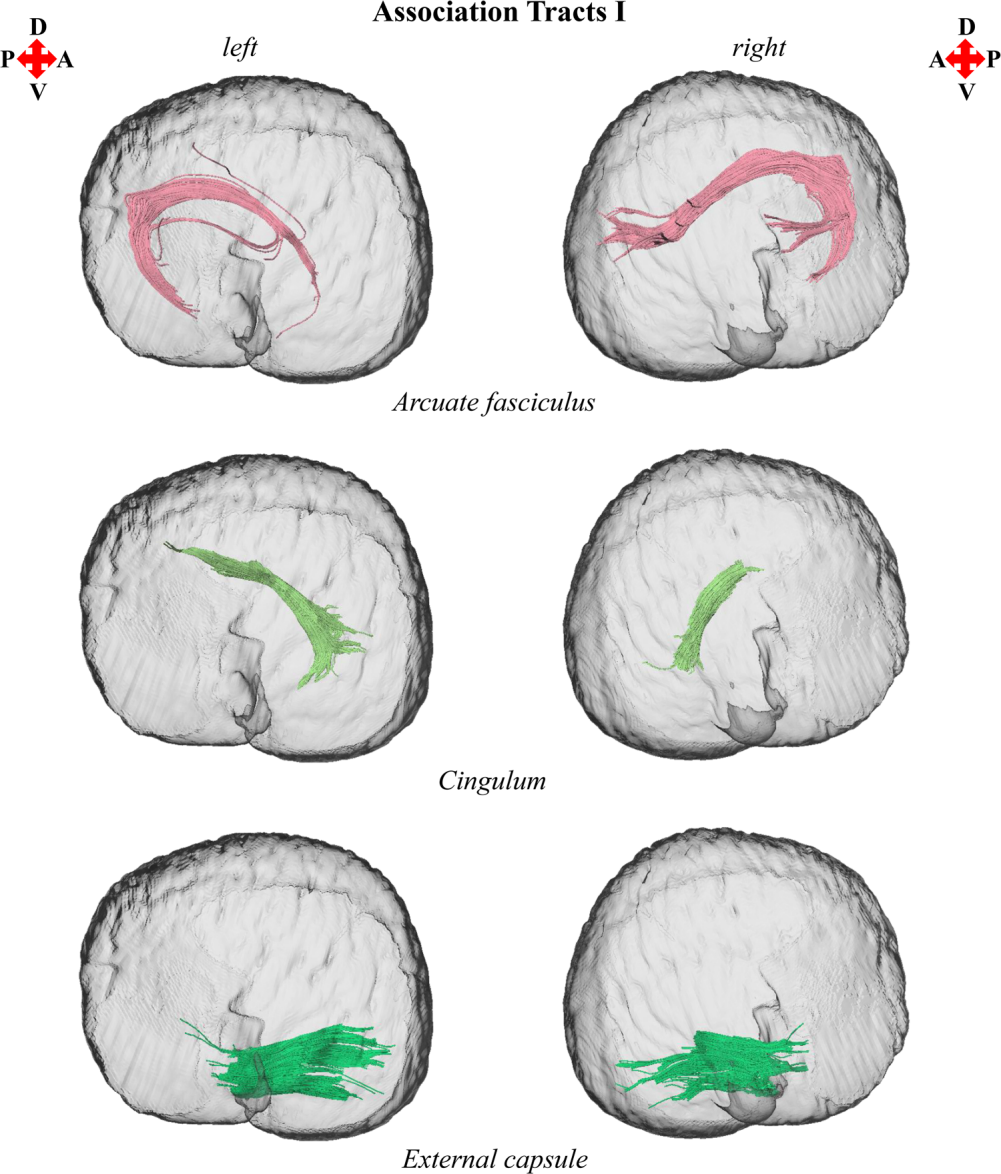
Left and right parasagittal views of the *T. truncatus* cerebral surface (translucent dark gray) and underlying white matter tracts of the association fiber system, including the arcuate fasciculus, cingulum, and external capsule. Color designations are consistent across figures

**Fig. 3:**
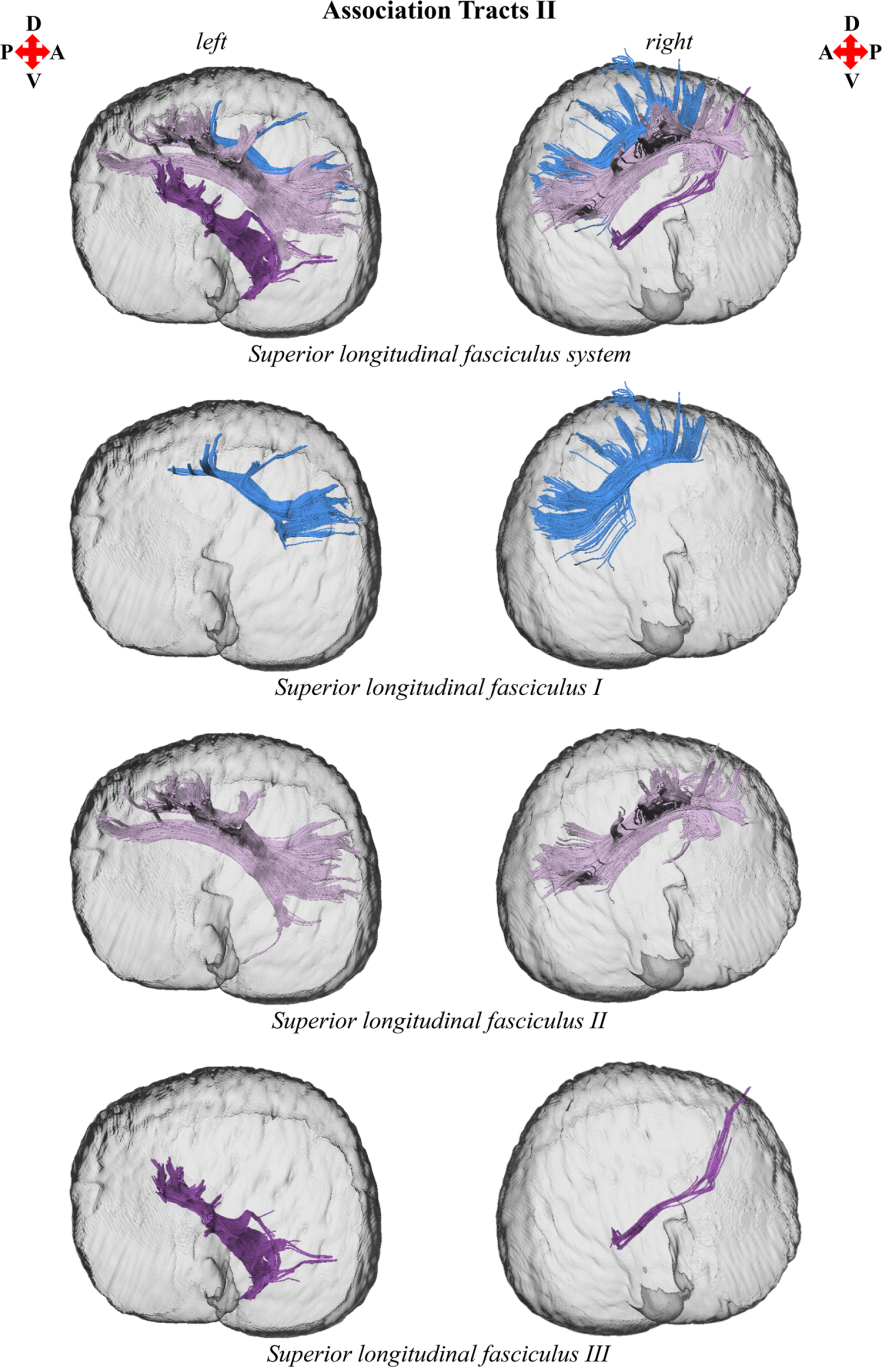
Left and right parasagittal views of the* T. truncatus* cerebral surface (translucent dark gray) and underlying associative superior longitudinal fasciculus system, comprising sub-tracts SLF I, SLF II, and SLF III. Color designations within the superior longitudinal fasciculus system reflect parcellation of sub-tracts SLF I, SLF II, and SLF III

**Fig. 4:**
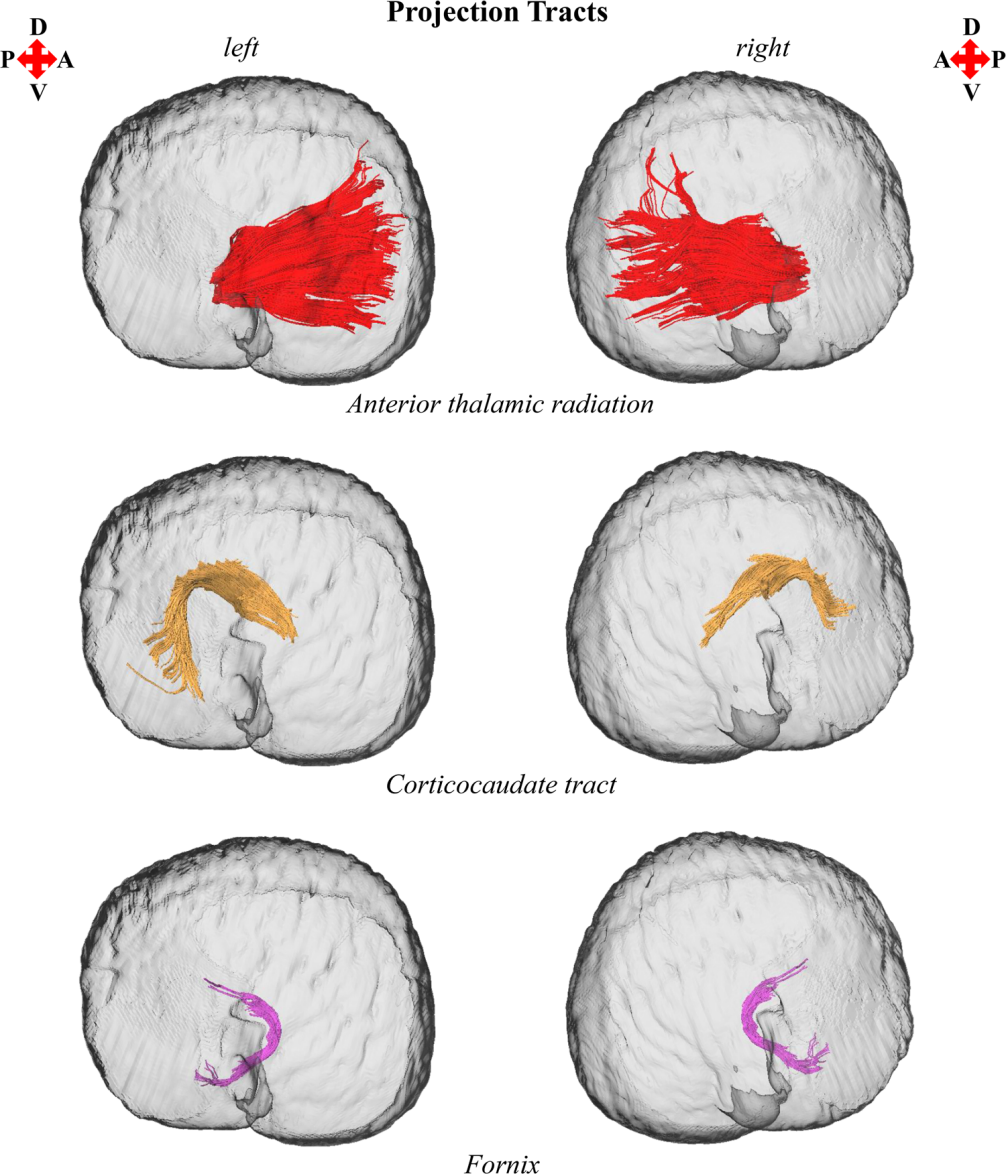
Left and right parasagittal views of the *T. truncatus* cerebral surface (translucent dark gray) and underlying white matter tracts of the projection fiber system, including the anterior thalamic radiation, corticocaudate tract, and fornix. Color designations are consistent across figures

**Fig. 5:**
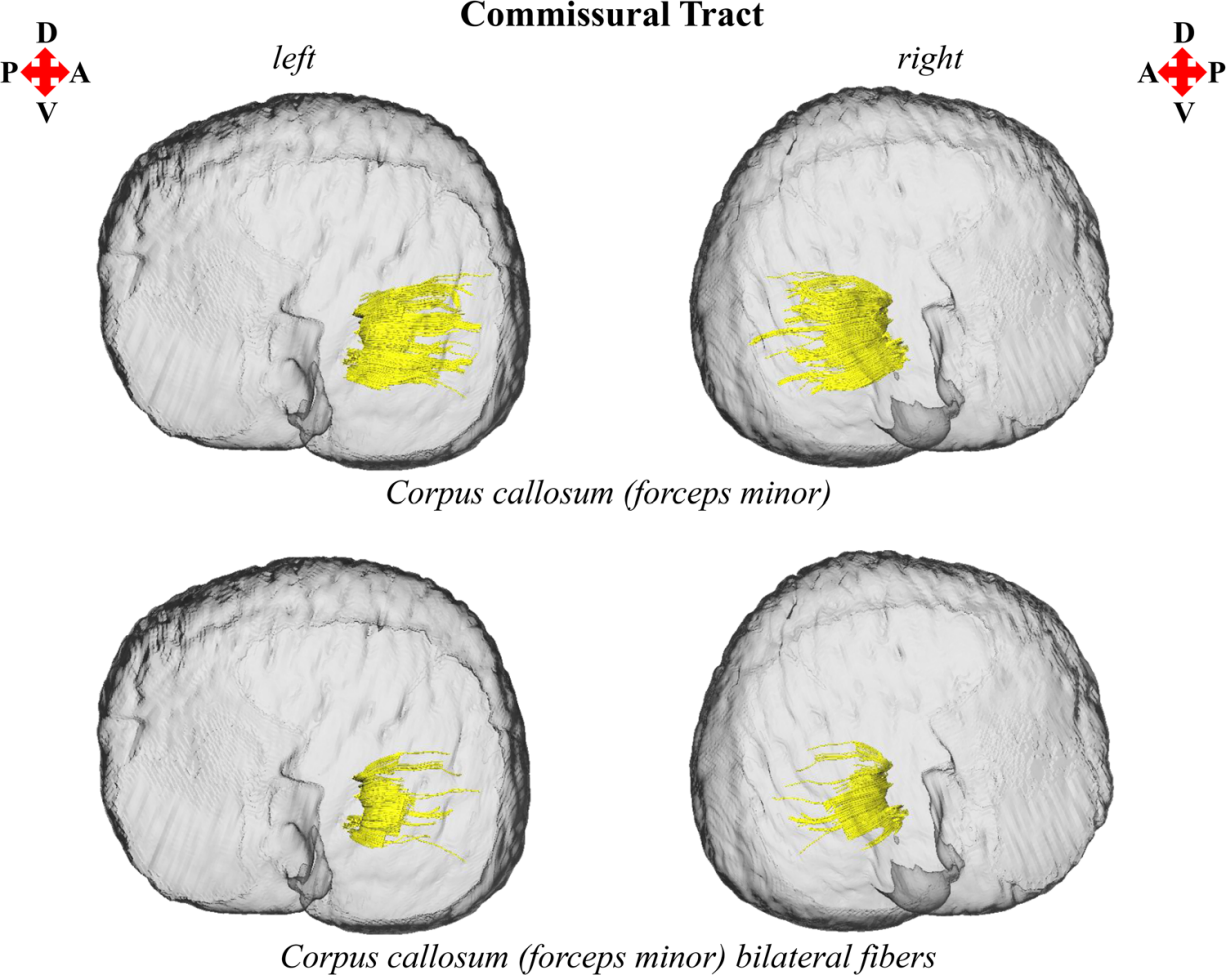
Left and right parasagittal views of the *T. truncatus* cerebral surface (translucent dark gray) and underlying corpus callosum of the commissural fiber system. Color designations are consistent across figures

3D reconstructions of the anterior thalamic radiation, arcuate fasciculus, cingulum, corticocaudate tract, external capsule, forceps minor of the corpus callosum, fornix, and superior longitudinal fasciculus system are shown in Figs. 1, 2, 3, 4, and 5. Reconstructions of the sub-tracts of the superior longitudinal fasciculus system (SLF I, SLF II, and SLF III) are displayed in Fig. 3. Tract-specific measurements (repeated measures mean ± standard deviation) of volume, fiber number, mean fiber length, FA, *M*_D_, *A*_D_, and *R*_D_ are provided in Online Resources 1 and 2.

**Fig. 6:**
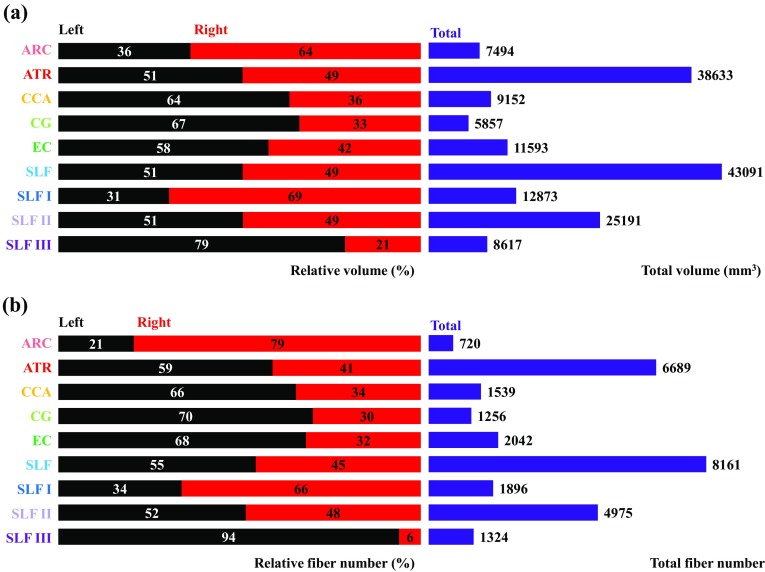
**a** Total volume (mm^3^, purple) and relative volume (%) for each tract (left, black; right, red) and **b** total fiber number (purple) and relative fiber number (%) for each tract (left, black; right, red). Left and right tracts combined represent 100% of the total volume or total fiber number. *ARC* arcuate fasciculus, *ATR* anterior thalamic radiation, *CCA* corticocaudate tract, *CG* cingulum, *EC* external capsule, *SLF* superior longitudinal fasciculus system, *SLF I* superior longitudinal fasciculus I, *SLF II* superior longitudinal fasciculus II, *SLF III* superior longitudinal fasciculus III

Asymmetries were found for the relative volumes of all of the tracts examined, with the exception of the anterior thalamic radiation, superior longitudinal fasciculus system, and sub-tract SLF II (Fig. 6a). Rightward asymmetry was observed for the relative volumes of the arcuate fasciculus and SLF I, whereas the corticocaudate tract, cingulum, external capsule, and SLF III were leftwardly asymmetric. Asymmetries in relative fiber number were observed for nearly all tracts and were generally greater in magnitude than the volumetric asymmetries (Fig. 6b). All of the asymmetric tracts examined exhibited a leftward bias in relative fiber number, except for the arcuate fasciculus and sub-tract SLF I, which were right lateralized. Pronounced lateralization of relative fiber number was observed for the right arcuate fasciculus and left SLF III, with each representing 79 and 94% of the total fiber number, respectively. Of all the tracts examined, the superior longitudinal fasciculus system and sub-tract SLF II were the only tracts to exhibit symmetry of relative fiber number.

**Fig. 7:**
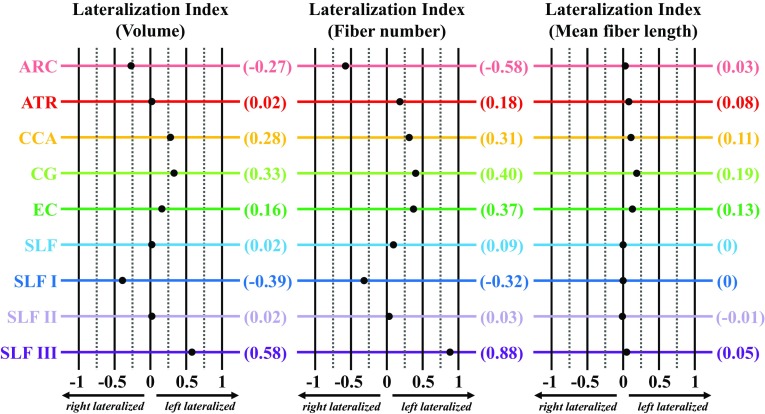
Lateralization index (LI) for the volume, fiber number, and mean fiber length of the arcuate fasciculus (ARC, rose), anterior thalamic radiation (ATR, red), corticocaudate tract (CCA, orange), cingulum (CG, light green), external capsule (EC, dark green), superior longitudinal fasciculus system (SLF, light blue), superior longitudinal fasciculus I (SLF I, dark blue), superior longitudinal fasciculus II (SLF II, light purple), and superior longitudinal fasciculus III (SLF III, dark purple). Tract-specific LI values for each measurement are shown in parentheses on the right. Color designations are consistent across figures; however, the superior longitudinal fasciculus system in Fig. 3 reflects parcellation of the sub-tracts, SLF I, SLF II, and SLF III

Lateralization indices for tract volume, fiber number, and mean fiber length are shown in Fig. 7. Volumetric LI values indicated leftward asymmetries for all of the white matter tracts, except for the arcuate fasciculus and sub-tract SLF I, which were right lateralized, and the anterior thalamic radiation, superior longitudinal fasciculus system, and sub-tract SLF II, which exhibited no asymmetry (−0.1 ≤ LI [Volume] ≤ 0.1). Fiber number LI values indicated asymmetry for all of the tracts examined, except for the superior longitudinal fasciculus system and sub-tract SLF II. Positive LI values for fiber number were observed for the anterior thalamic radiation, corticocaudate tract, cingulum, external capsule, and SLF III, whereas the rightwardly asymmetric arcuate fasciculus and SLF I exhibited negative LI values. LI values corresponding to fiber number were greater than LI values for tract volume for all of the asymmetrical tracts, excluding sub-tract SLF I. Asymmetry of mean fiber length was less widespread than that of volume and fiber number, with only a third of the tracts demonstrating lateralization. Of the six major tracts examined, only the superior longitudinal fasciculus system exhibited consistent symmetry of LI values across tract-specific measurements of volume, fiber number, and mean fiber length. However, parcellation of the superior longitudinal fasciculus system into its subcomponents revealed pronounced lateralization of SLF I and SLF III volumes and fiber numbers. The preponderance of asymmetrical tracts and sub-tracts were left lateralized (Fig. 7).

There was an absence of lateralization (−0.1 ≤ LI [*X*] ≤ 0.1) for the measurements of FA, *M*_D_, *A*_D_, and *R*_D_ in all of the tracts examined (Online Resource 3), which suggests that the tract-specific measurements of volume, fiber number, and mean fiber length were not confounded by these parameters and were indeed asymmetric in certain tracts. Moreover, symmetry of microstructural diffusion parameters and uniformity of the anatomical datasets indicate that macrostructural asymmetries were not due to tissue damage or incomplete fixation of the specimen.

## Discussion

The present study represents the first investigation of cerebral white matter asymmetry in a cetacean. Given the difficulty of obtaining cetacean specimens, particularly those of suitable quality for DTI analysis, only one *T. truncatus* brain was included in this investigation. Based on its species-appropriate brain mass (Pilleri and Gihr 1970; Tarpley and Ridgway 1994; Marino 1998) and comparatively normal appearance on structural MR images (Marino et al. 2001; Ridgway et al. 2006b; Hanson et al. 2013), it is not suspected that the macrostructural white matter asymmetry observed in this *T. truncatus* specimen was anomalous. Moreover, the demonstration of stability of macro- and microstructural white matter asymmetry in *H. sapiens* with aging (Takao et al. 2010, 2013; Stamatakis et al. 2011; but cf. Ardekani et al. 2007) suggests that the structural asymmetries, or lack thereof, observed in this 27-year-old *T. truncatus* brain were not associated with senescence. However, future DTI studies are required to examine *T. truncatus* specimens of varying age, sex, and ecotype to increase confidence in the results of this study and their interpretation.

The findings of this investigation suggest widespread structural asymmetries of cerebral white matter in this *T. truncatus* and provide support for the hypothesis that large brains should exhibit pronounced lateralization (Ringo 1991; Ringo et al. 1994). Moreover, the sparse reconstruction of the corpus callosum in this *T. truncatus* (Figs. 1, 5) in parallel with various reports on the diminutive size of the cetacean corpus callosum relative to the volume of the cerebral hemispheres (Tarpley and Ridgway 1994; Keogh and Ridgway 2008; Montie et al. 2008; Manger et al. 2010; Berns et al. 2015; Wright et al. 2017) correspond to observations and predictions of reduced interhemispheric connectivity with brain enlargement (Ringo 1991; Ringo et al. 1994; Rilling and Insel 1999; Olivares et al. 2000, 2001). In addition, it is plausible that distinctive structural scaling and selective pressures of the aquatic environment have also contributed to white matter asymmetry in *T. truncatus* and potentially other members of Cetacea. To address constraints on neuronal interconnectivity and transmission times associated with increased brain size, a unique cerebral scaling strategy that could further maximize brain lateralization may have been selected for in* T. truncatus* and other members of the cetacean superfamily Delphinoidea. Whereas all other mammals exhibit allometric scaling of cerebral white matter (i.e., a disproportionate expansion of white matter compared to gray matter; Barton and Harvey 2000; Zhang and Sejnowski 2000), the cerebral white matter of delphinoids scales isometrically with increasing brain size (Hofman 1989; Wright et al. 2017). Mammalian white matter hyperscaling is thought to arise from the need for thicker axons to increase conduction velocity in large brains that have greater interneuronal distances and consequently, require longer axonal connections (Chklovskii and Stevens 2000; Zhang and Sejnowski 2000; Changizi 2001); however, disproportionate expansion of white matter is insufficient to maintain complete neuronal interconnectivity and overcome significant transmission delays, potentially promoting the clustering of related functions and ultimately, brain lateralization (Ringo 1991; Ringo et al. 1994; Changizi 2001). Without the compensatory mechanism of white matter hyperscaling, it may be suggested that the brains of delphinoids, and potentially other cetaceans, would be characterized by fewer connections and greater asymmetry than expected for a brain of the same size subject to typical mammalian allometric scaling. Moreover, the demands of an aquatic existence may necessitate continuous vigilance resulting in an extreme form of functional lateralization, unihemispheric slow wave sleep (Lyamin et al. 2008; Branstetter et al. 2012), potentially supported by the reduced interhemispheric connectivity (Figs. 1, 5; Tarpley and Ridgway 1994) and prevalent intrahemispheric white matter asymmetries revealed in this study (Figs. 2, 3, 4, 6, 7).

The cerebral white matter asymmetry reported for this *T. truncatus* complements previous evidence for structural, functional, and behavioral lateralization in Cetacea. Furthermore, examination of prior investigations in light of the findings of this study may provide insights into the functional significance of the structural lateralization observed. Along with neuroanatomical asymmetries of cortical surface area (Ridgway and Brownson 1984), gray matter volume (Montie et al. 2008; Wright et al. 2017), and intrahemispheric white matter volume and fiber number, delphinids and the wider Odontoceti exhibit varying degrees of asymmetry of the surrounding cranium and epicranial complex (i.e., an assemblage of nasal structures responsible for acoustic signal generation; Ness 1967; Cranford et al. 1996). Odontocete cranial and epicranial asymmetry may be related to the evolution of echolocation; however, it may alternatively be associated with laryngeal asymmetry facilitating prey ingestion (MacLeod et al. 2007) or directional hearing in water (Renaud and Popper 1975; Branstetter and Mercado 2006; Fahlke et al. 2011). Moreover, it has been proposed that epicranial asymmetry may facilitate the production of complex and diverse acoustic signals and cause marked lateralization of emitted sounds (Cranford et al. 1996; Huggenberger et al. 2010; Frainer et al. 2015). Relevant to this interpretation is the demonstration of directional bias for the production of functionally distinct acoustic signals by delphinoids, including the *T. truncatus* of the present study (NAY; Ridgway et al. 2009), with their independently and simultaneously operable phonic lips (i.e., sound generators; Cranford et al. 2011; Ridgway et al. 2015). The delphinoids *T. truncatus*, *Pseudorca crassidens*, and *Phocoena phocoena* demonstrate a preference for emitting echolocation signals (i.e., predominantly nonsocial high-frequency broadband clicks) from the right pair of phonic lips (Ridgway et al. 2009; Madsen et al. 2010, 2013). Moreover, *T. truncatus* and *P. crassidens* exhibit a preference for emitting communication signals (i.e., social lower frequency whistles) from the left pair of phonic lips (Ridgway et al. 2009; Madsen et al. 2013). Since the presentation of auditory as well as visual and somatosensory stimuli evokes larger responses in the contralateral cerebral hemisphere in delphinoids (Bullock et al. 1968; Bullock and Ridgway 1972; Supin et al. 1978; Ridgway and Carder 1990; Ridgway et al. 2015), the directional emittance of behaviorally distinct sounds could indicate differential processing by the right hemisphere for social communicative information and left hemisphere for nonsocial echolocative information. Specifically, the returning echoes of high-frequency clicks generated by the right pair of phonic lips, which are presumably controlled by the left hemisphere, should reach the ipsilateral jaw first leading to earlier processing of echolocation information by the contralateral left hemisphere; whereas, perception of lower frequency whistles produced by the left pair of phonic lips, which are presumably controlled by the right hemisphere, should occur more rapidly with the ipsilateral jaw leading to earlier processing of communication signals in the contralateral right hemisphere. This auditory schema for the asymmetric production, perception, and processing of acoustic signals of differing frequencies by delphinoids finds support in the double filtering by frequency (DFF) theory proposed by Ivry and Robertson (1998). DFF theory is partially based on pitch perception experiments which indicate a left hemisphere bias for processing relatively high-frequency sounds and a right hemisphere bias for processing relatively low-frequency sounds (Ivry and Lebby 1993; Ivry and Robertson 1998). The pervasive white matter asymmetry of this *T. truncatus* (Figs. 2, 3, 4, 6, 7) may underpin this proposed functional lateralization of frequency processing and the lateralized production of high-frequency echolocation clicks and lower frequency communication whistles observed in vita (NAY; Ridgway et al. 2009).

Of relevance to the proposed lateralized processing of lower frequency communication signals in *T. truncatus* is the arcuate fasciculus (Figs. 1, 2). In anthropoid primates, the arcuate fasciculus connects the frontal, parietal, and temporal lobes (Catani and Thiebaut de Schotten 2008; Rilling et al. 2008). In *H. sapiens*, arcuate terminations include Broca’s territory (i.e., speech production site), Wernicke’s area (i.e., speech comprehension site), and proximal areas (e.g., middle and inferior temporal gyri; Catani et al. 2005, 2007; Rilling et al. 2008). Moreover, the arcuate fasciculus of the primates *Pan troglodytes* and *Macaca mulatta* connects homologs of Broca’s and Wernicke’s areas (Catani et al. 2005; Rilling et al. 2008; Rilling 2014) associated with the production of orofacial expressions (Petrides et al. 2005) and communicative signals (i.e., gestural and vocal signaling; Taglialatela et al. 2008) as well as the perception of conspecific vocalizations (Gil-da-Costa et al. 2006; Taglialatela et al. 2009). Compared to nonhuman primates, the arcuate fasciculus of *H. sapiens* is considerably different exhibiting unique structural elaboration and cortical terminations thought to be associated with the evolution of language (Rilling et al. 2008). In *H. sapiens* and *P. troglodytes*, the arcuate fasciculus is predominantly left lateralized (Catani et al. 2007; Glasser and Rilling 2008; Thiebaut de Schotten et al. 2011; Rilling et al. 2012; Fernández-Miranda et al. 2014) potentially indicating a left hemisphere specialization for species-specific communication. In contrast, the arcuate fasciculus of this *T. truncatus* exhibited pronounced rightward asymmetry (Figs. 2, 6, 7). If the arcuate terminations of *T. truncatus* are functionally homologous to those of primates, then this finding may suggest a right hemisphere bias for conspecific communication in agreement with behavioral observations demonstrating directional bias for the production of social acoustic signals (i.e., lower frequency whistles; Ridgway et al. 2009; Madsen et al. 2013). Interestingly, the right hemisphere bias for conspecific communication proposed for *T. truncatus* contrasts with evidence for left lateralization of communicative functions in nearly all other mammals studied to date (Ocklenburg et al. 2013). A recent DTI study identified a direct auditory pathway from the inferior colliculus to the ipsilateral temporal lobe in the delphinids, *Delphinus delphis* and *Stenella attenuata* (Berns et al. 2015); however, structural asymmetry of this pathway was not assessed in that study, nor could it be evaluated in the present study due to susceptibility artifacts in the data localized in the brainstem. It would be of interest in future DTI investigations of *T. truncatus* and other cetaceans to compare the lateralization of this direct auditory pathway to that of the arcuate fasciculus. In addition to the proposed functional lateralization of social acoustic signals, arcuate asymmetry may also be relevant to accumulating reports of behavioral lateralization in delphinoids regarding visual (Karenina et al. 2010; Thieltges et al. 2011; Karenina et al. 2013a, b; Yeater et al. 2014), somatosensory, and motor (Johnson and Moewe 1999; Sakai et al. 2006; Hill et al. 2015) social signaling.

Regarding the proposed left hemisphere bias for the perception, processing, and production of nonsocial echolocation signals (i.e., high-frequency clicks), it is interesting to note that in this *T. truncatus* the majority of bilateral tracts were left lateralized. Increased tract size could reflect certain biophysical properties of axons, specifically greater axonal diameter, axon abundance, or degree of myelination. Increases in axon diameter and myelination are factors associated with increased conduction velocity and enlarged axonal volume (Hursh 1939; Waxman 1980; Wang et al. 2008). Moreover, studies showing a greater prevalence of myelinated axons and large myelinated axons (diameter >2 µm) with brain enlargement suggest that these changes in white matter architecture serve to reduce conduction delays arising from increased transmission distances across large brains (Wang et al. 2008; Caminiti et al. 2009). Since white matter volume is positively associated with biophysical axonal properties (i.e., axon diameter and myelination), which, in turn, positively correlate with conduction velocity, it is plausible that tract asymmetry reflects conduction velocity differences between the cerebral hemispheres. However, the symmetries of microstructural diffusion parameters (i.e., FA, *M*_D_, *A*_D_, and *R*_D_) found for all tracts and sub-tracts in this *T. truncatus* do not clarify which axonal properties influenced tract asymmetry. Thus, future histological studies are needed to determine the extent to which asymmetrical tract volumes reflect differences in axonal diameter, abundance, or myelination in cetaceans. Ultimately, the preponderance of enlarged tracts in the left cerebral hemisphere of this *T. truncatus* (Figs. 2, 3, 4, 6, 7) could reflect a requirement for rapid analysis of high-frequency echolocation signals transmitted within an aquatic medium that quadruples sound velocity. Widespread leftward structural asymmetries along with the lateralized production of echolocation clicks by the right pair of phonic lips (Ridgway et al. 2009; Madsen et al. 2010, 2013; Ridgway et al. 2015) suggest a left hemisphere bias for nonsocial echolocative functions. Moreover, the cetacean left hemisphere has previously been implicated in predatory locomotor activity. Odontocetes and mysticetes both exhibit rightward action biases during foraging behaviors, including strand, mud plume, and lunge feeding, fish chasing and herding, and rolling during feeding dives (MacNeilage 2013; Karenina et al. 2016). The observation of largely left-lateralized tracts in this *T. truncatus* may be related to both echolocative function and the strong rightward action asymmetries observed in Cetacea, allowing for rapid and responsive perception and pursuit of prey. Interestingly, *H. sapiens* and *P. troglodytes* also have more leftward than rightward cerebral white matter asymmetries (Pujol et al. 2002; Wakana et al. 2007; Cantalupo et al. 2009; Hopkins et al. 2010). Although its functional significance in *H. sapiens* is unclear, the preponderance of leftward white matter asymmetries in *P. troglodytes* has been suggested to relate to evidence for task-specific population-level right-hand usage (Lonsdorf and Hopkins 2005; Cantalupo et al. 2009; Hopkins et al. 2010), potentially providing comparative support for the proposed association between predominantly left-lateralized tracts in *T. truncatus* and observed rightward foraging biases in cetaceans.

Brain enlargement, isometric cerebral white matter scaling, and the unique demands of the aquatic environment may each potentially contribute to the widespread cerebral white matter asymmetries observed in the present study of the *T. truncatus* brain. As the first investigation of cetacean white matter asymmetry, this study provides a heretofore undescribed neuroanatomical basis for functional and behavioral lateralization in* T. truncatus* and potentially other cetaceans. Reviewing the available literature, pervasive asymmetry of white matter architecture is tentatively proposed to reflect lateralization of social and nonsocial sensory and motor functions. Moreover, detection of a right-lateralized arcuate fasciculus raises interesting and important questions about the nature of cetacean communication and the plasticity of hemispheric specialization. Future DTI and functional MRI (fMRI) studies of *T. truncatus* and other cetaceans are needed to characterize cerebral white matter asymmetry across a wide range of individuals and species, and more specifically, to establish the predominant directionality of arcuate lateralization and elucidate the function of arcuate cortical terminations. The growing availability of wide-bore MRI systems capable of accommodating larger animals may facilitate future fMRI studies of delphinids. With proper preparation,* T. truncatus* can be trained to slide out of the water and lie in a scanner (Ridgway et al. 2006b). Moreover, *T. truncatus* can echolocate while out of water (Finneran et al. 2010). With such animals and imaging equipment, great progress can be made in understanding the organization and function of the cetacean brain.

### Electronic supplementary material

Below is the link to the electronic supplementary material.

Online Resource 1 (PDF 8 kb)

Online Resource 2 (PDF 9 kb)

**Online Resource 3** Lateralization index (LI) for the fractional anisotropy (FA), mean diffusivity (*M*_D_), axial diffusivity(*A*_D_), and radial diffusivity (*R*_D_) of the arcuate fasciculus (ARC, *rose*), anterior thalamic radiation (ATR, *red*), corticocaudate tract (CCA, *orange*), cingulum (CG, *light green*), external capsule (EC, *dark green*), superior longitudinal fasciculus system (SLF, *light blue*), superior longitudinal fasciculus I (SLF I, *dark blue*), superior longitudinal fasciculus II (SLF II, *light purple*), and superior longitudinal fasciculus III (SLF III, *dark purple*). Tract-specific LI values for each measurement are shown in parentheses on the right. Color designations are consistent across figures; however, the superior longitudinal fasciculus system in Fig. 3 reflects parcellation of the sub-tracts, SLF I, SLF II, and SLF III (PDF 17 kb)

## Appendix: ROI protocols

The reconstruction protocols for each tract of interest involved different permutations of three DtiStudio operations, OR, AND, or NOT (Jiang et al. 2006; Wakana et al. 2007). The OR operation permitted the initial selection of fibers with the placement of the first ROI on an anatomical landmark. Placement of a second ROI with the AND operation restricted the selected fibers to those that penetrated both the first and second ROIs. ROIs applied with the NOT operation removed the circumscribed subset of fibers from the previously selected tract.

### Arcuate fasciculus

In mid-sagittal view, select the coronal slice 18 mm anterior to the splenium of the corpus callosum. In this coronal slice, draw the first ROI (OR operation) around the superior longitudinal fasciculus core and branches of the suprasylvian, ectosylvian, and perisylvian gyri, located dorsolateral to the internal capsule. Place a second ROI (AND operation) around the temporally-projecting fibers of the arcuate fasciculus on the axial slice 21 mm dorsal to the genu of the corpus callosum when viewed mid-sagittally.

### Anterior thalamic radiation

In mid-sagittal view, select the coronal slice 12 mm anterior to the splenium of the corpus callosum. In this coronal slice, draw the first ROI (OR operation) around the entire thalamus. Draw a second ROI (AND operation) around the anterior limb of the internal capsule in the coronal slice at the level of the anteriormost portion of the genu of the corpus callosum when viewed mid-sagittally. Place subsequent ROIs (NOT operation) to remove: (a) fibers extending to the contralateral hemisphere; (b) fibers extending ventrally and posteriorly from the thalamus; (c) callosal fibers; (d) corticocaudate fibers; and (e) stray fibers.

### Cingulum

In mid-sagittal view, select the coronal slice at the level of the anteriormost portion of the genu of the corpus callosum. In this coronal slice, draw the first ROI (OR operation) around the cingulum, located dorsal to the corpus callosum. Place a second ROI (AND operation) around the cingulum on the coronal slice 15 mm posterior to the genu of the corpus callosum (near the callosal midpoint) when viewed mid-sagittally. Draw subsequent ROIs (NOT operation) to remove: (a) callosal fibers; and (b) stray fibers.

### Corticocaudate tract

In mid-sagittal view, select the axial slice at the level of the dorsalmost portion of the body of the corpus callosum. In this axial slice, draw the first ROI (OR operation) around the caudate nucleus. Place a second ROI (AND operation) around the caudate nucleus in the coronal slice at the level of the mid-splenium of the corpus callosum when viewed mid-sagittally. Place subsequent ROIs (NOT operation) to remove: (a) anterior fibers extending ventrally; (b) callosal fibers; (c) corona radiata fibers; (d) internal capsule fibers; (e) thalamic fibers; and (f) stray fibers.

### External capsule

In mid-sagittal view, select the coronal slice at the level of the anteriormost portion of the fornix. In this coronal slice, draw the first ROI (OR operation) around the external capsule, located lateral to the internal capsule. Place subsequent ROIs (NOT operation) to remove: (a) fibers extending to the contralateral hemisphere; (b) internal capsule fibers; and (c) stray fibers.

### Forceps minor of the corpus callosum

In mid-sagittal view, select the coronal slice at the level of the anteriormost portion of the genu of the corpus callosum. In this coronal slice, draw the first ROI (OR operation) around the genu of the corpus callosum. The callosal genu and first ROI exhibit a distinct butterfly shape. Place subsequent ROIs (NOT operation) to remove: (a) fibers extending posteriorly from the genu; (b) cingulum fibers; (c) internal capsule fibers; (d) obvious non-bilaterally projecting fibers apparent in the anterior and ventral portions of the forceps minor; and (e) stray fibers. In order to isolate the bilaterally projecting fibers of the forceps minor, place a final ROI (AND operation) around the genu of the corpus callosum when viewed mid-sagittally.

### Fornix

In mid-sagittal view, select the coronal slice 3 mm posterior to the anteriormost portion of the fornix. In this coronal slice, draw the first ROI (OR operation) around the fornix, located ventral to the corpus callosum and septum pellucidum tract. Place subsequent ROIs (NOT operation) to remove: (a) fibers medial to the anterior columns of the fornix; (b) callosal fibers; and (c) septum pellucidum tract fibers.

### Superior longitudinal fasciculus

In mid-sagittal view, select the coronal slice 18 mm anterior to the splenium of the corpus callosum. In this coronal slice, draw the first ROI (OR operation) around the superior longitudinal fasciculus core and branches of the suprasylvian, ectosylvian, and perisylvian gyri, located dorsolateral to the internal capsule. Place a second ROI (AND operation) around the superior longitudinal fasciculus core and branches in the coronal slice at the level of the anteriormost portion of the genu of the corpus callosum when viewed mid-sagittally. Place subsequent ROIs (NOT operation) to remove: (a) arcuate fasciculus fibers; (b) external capsule fibers; (c) internal capsule fibers; (d) lateral gyrus fibers; and (e) stray fibers.

In order to isolate the subcomponents of the superior longitudinal fasciculus system (SLF I, SLF II, and SLF III), placement of additional ROIs on the coronal slice 18 mm anterior to the splenium of the corpus callosum is required. For SLF I, an ROI (AND operation) was drawn around the white matter of the suprasylvian gyrus. For SLF II, an ROI (AND operation) was drawn around the white matter of the ectosylvian gyrus. Due to the lack of an apparent demarcation between the SLF I and SLF II within the superior longitudinal fasciculus core, an arbitrary line was drawn from the lateralmost base of the SLF I branch to the dorsalmost portion of the lateral ventricle to separate SLF I and SLF II core fibers. An ROI (AND operation) was drawn around the white matter of the perisylvian gyrus to isolate SLF III.
