# Supplementary material for: Diffusion tractography reveals pervasive asymmetry of cerebral white matter tracts in the bottlenose dolphin (Tursiops truncatus)
Source: Brain Struct Funct. 2017 Nov 30;223(4):1697–711. doi: 10.1007/s00429-017-1525-9 (PMC5884918; doi:10.1007/s00429-017-1525-9)
Supplement: Supplementary file 1 — Online Resource 1 (PDF 8 kb) [file 429_2017_1525_MOESM1_ESM.pdf]

Online Resource 1 Repeated measures mean and standard deviation (± SD) for macrostructural tract-specific parameters of volume, fiber number, and mean fiber length in *T. truncatus* (N=1)

Tracts		Volume (mm^3) *Mean ± SD*	Fiber number *Mean ± SD*	Mean fiber length (mm) *Mean ± SD*
**ARC**				
	Left	2734 ± 0	150 ± 0	110 ± 0
	Right	4760 ± 0	570 ± 0	105 ± 0
**ATR**				
	Left	19653 ± 1298	3955 ± 355	60.0 ± 0.5
	Right	18980 ± 278	2734 ± 75	50.9 ± 0.1
**CCA**				
	Left	5839 ± 149	1009 ± 68	45.3 ± 1.4
	Right	3313 ± 241	530 ± 70	36.5 ± 1.7
**CCFM**				
	**CCFM**	10894 ± 168	2321 ± 56	25.1 ± 0.3
	**CCFMBi**	4775 ± 93	807 ± 56	31.0 ± 0.6
**CG**				
	Left	3895 ± 5	882 ± 1	57.7 ± 0
	Right	1962 ± 31	374 ± 6	39.1 ± 0.1
**EC**				
	Left	6727 ± 305	1396 ± 71	40.9 ± 0.9
	Right	4866 ± 248	646 ± 52	31.5 ± 0.5
**FX**				
		2355 ± 96	386 ± 27	38.2 ± 1.4
**SLF**				
	Left	21924 ± 0	4450 ± 0	65.2 ± 0
	Right	21167 ± 124	3711 ± 36	65.0 ± 0.2
	**SLF I**			
	Left	3938 ± 330	645 ± 73	56.4 ± 1.2
	Right	8935 ± 556	1251 ± 68	56.2 ± 0.1
	**SLF II**			
	Left	12838 ± 168	2573 ± 63	68.6 ± 0
	Right	12353 ± 434	2402 ± 67	69.7 ± 0.3
	**SLF III**			
	Left	6818 ± 0	1245 ± 0	62.6 ± 0
	Right	1799 ± 0	79.0 ± 0	56.8 ± 0

ARC (arcuate fasciculus), ATR (anterior thalamic radiation), CCA (corticocaudate tract), CCFM (corpus callosum - forceps minor), CCFMBi (corpus callosum - forceps minor, bilateral fibers), CG (cingulum), EC (external capsule), FX (fornix), SLF (superior longitudinal fasciculus system), SLF I (superior longitudinal fasciculus I), SLF II (superior longitudinal fasciculus II), SLF III (superior longitudinal fasciculus III)
