# Supplementary material for: Diffusion tractography reveals pervasive asymmetry of cerebral white matter tracts in the bottlenose dolphin (Tursiops truncatus)
Source: Brain Struct Funct. 2017 Nov 30;223(4):1697–711. doi: 10.1007/s00429-017-1525-9 (PMC5884918; doi:10.1007/s00429-017-1525-9)
Supplement: Supplementary file 2 — Online Resource 2 (PDF 9 kb) [file 429_2017_1525_MOESM2_ESM.pdf]

Online Resource 2 Repeated measures mean and standard deviation (± SD) for microstructural tract-specific parameters of fractional anisotropy (FA), mean diffusivity (M_D), axial diffusivity (A_D), and radial diffusivity (R_D) in *T. truncatus* (N=1)

**Tracts**		**FA** *Mean ± SD*	**M_D (mm^2/s)** *Mean ± SD*	**A_D (mm^2/s)** *Mean ± SD*	**R_D (mm^2/s)** *Mean ± SD*
**ARC**					
	Left	0.20 ± 0	1.91E-04 ± 0	2.31E-04 ± 0	1.70E-04 ± 0
	Right	0.21 ± 0	1.87E-04 ± 0	2.29E-04 ± 0	1.67E-04 ± 0
**ATR**					
	Left	0.28 ± 0	2.04E-04 ± 0	2.63E-04 ± 0	1.74E-04 ± 1.04E-06
	Right	0.28 ± 0	2.25E-04 ± 0	2.92E-04 ± 0	1.91E-04 ± 0
**CCA**					
	Left	0.24 ± 0	2.74E-04 ± 2.52E-06	3.43E-04 ± 2.32E-06	2.40E-04 ± 2.62E-06
	Right	0.23 ± 0	2.62E-04 ± 2.54E-06	3.25E-04 ± 3.23E-06	2.30E-04 ± 2.14E-06
**CCFM**					
	**CCFM**	0.32 ± 0	2.12E-04 ± 0	2.85E-04 ± 0	1.76E-04 ± 0
	**CCFMBi**	0.32 ± 0	2.08E-04 ± 0	2.81E-04 ± 0	1.72E-04 ± 0
**CG**					
	Left	0.23 ± 0	2.16E-04 ± 0	2.71E-04 ± 0	1.89E-04 ± 0
	Right	0.23 ± 0	2.26E-04 ± 0	2.83E-04 ± 0	1.97E-04 ± 0
**EC**					
	Left	0.28 ± 0	2.51E-04 ± 1.80E-06	3.26E-04 ± 2.50E-06	2.14E-04 ± 1.50E-06
	Right	0.23 ± 0	2.83E-04 ± 1.70E-06	3.53E-04 ± 2.23E-06	2.48E-04 ± 1.50E-06
**FX**					
		0.18 ± 0	3.35E-04 ± 0	3.99E-04 ± 0	3.03E-04 ± 0
**SLF**					
	Left	0.22 ± 0	1.80E-04 ± 0	2.21E-04 ± 0	1.60E-04 ± 0
	Right	0.22 ± 0	1.72E-04 ± 0	2.14E-04 ± 0	1.51E-04 ± 0
	**SLF I**				
	Left	0.22 ± 0	1.84E-04 ± 0	2.29E-04 ± 1.15E-06	1.62E-04 ± 0
	Right	0.23 ± 0	1.65E-04 ± 0	2.05E-04 ± 0	1.45E-04 ± 0
	**SLF II**				
	Left	0.23 ± 0	1.75E-04 ± 0	2.18E-04 ± 0	1.54E-04 ± 0
	Right	0.23 ± 0	1.74E-04 ± 0	2.17E-04 ± 0	1.53E-04 ± 0
	**SLF III**				
	Left	0.18 ± 0	1.88E-04 ± 0	2.23E-04 ± 0	1.70E-04 ± 0
	Right	0.18 ± 0	2.03E-04 ± 0	2.42E-04 ± 0	1.84E-04 ± 0

ARC (arcuate fasiculus), ATR (anterior thalamic radiation), CCA (corticocaudate tract), CCFM (corpus callosum - forceps minor), CCFMBi (corpus callosum - forceps minor, bilateral fibers), CG (cingulum), EC (external capsule), FX (fornix), SLF (superior longitudinal fasciculus system), SLF I (superior longitudinal fasciculus I), SLF II (superior longitudinal fasciculus II), SLF III (superior longitudinal fasciculus III)
