# Supplementary figures and images for: Diffusion tractography reveals pervasive asymmetry of cerebral white matter tracts in the bottlenose dolphin (Tursiops truncatus)
Source: Brain Struct Funct. 2017 Nov 30;223(4):1697–711. doi: 10.1007/s00429-017-1525-9 (PMC5884918; doi:10.1007/s00429-017-1525-9)

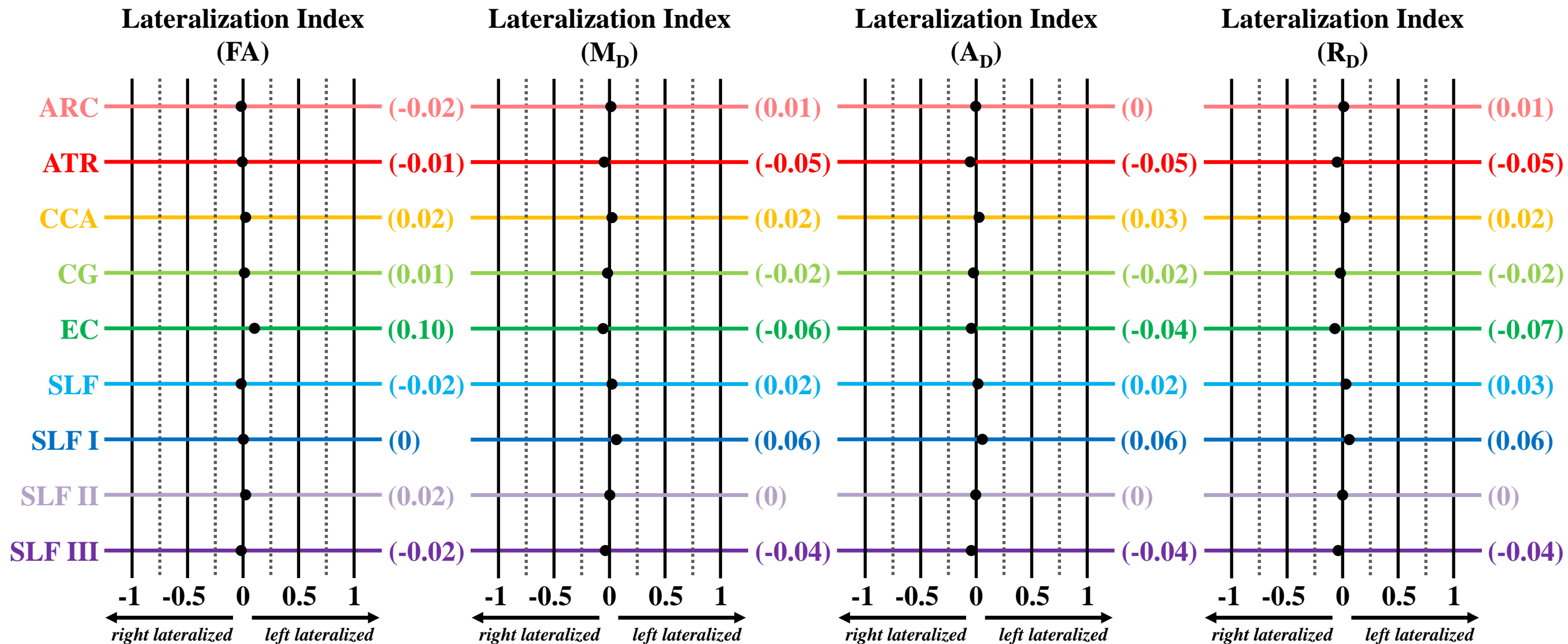

Supplement: Supplementary file 3 — Online Resource 3 Lateralization index (LI) for the fractional anisotropy (FA), mean diffusivity (M D), axial diffusivity(A D), and radial diffusivity (R D) of the arcuate fasciculus (ARC, rose), anterior thalamic radiation (ATR, red), corticocaudate tract (CCA, orange), cingulum (CG, light green), external capsule (EC, dark green), superior longitudinal fasciculus system (SLF, light blue), superior longitudinal fasciculus I (SLF I, dark blue), superior longitudinal fasciculus II (SLF II, light purple), and superior longitudinal fasciculus III (SLF III, dark purple). Tract-specific LI values for each measurement are shown in parentheses on the right. Color designations are consistent across figures; however, the superior longitudinal fasciculus system in Fig. 3 reflects parcellation of the sub-tracts, SLF I, SLF II, and SLF III (PDF 17 kb) [file 429_2017_1525_MOESM3_ESM.pdf]
